# Outcomes of cervical spinal stenosis surgery in patients aged ≥ 65 years based on insurance status: a single-center cohort study from a tertiary center in Germany

**DOI:** 10.1007/s00701-023-05700-9

**Published:** 2023-07-06

**Authors:** Pavlina Lenga, Vassilios Papakonstantinou, Karl Kiening, Andreas W. Unterberg, Basem Ishak

**Affiliations:** Department of Neurosurgery, Heidelberg University Hospital, Im Neuenheimer Feld 400, 69120 Heidelberg, Germany

**Keywords:** Insurance, Myelopathy, Surgery

## Abstract

**Objective:**

The prevalence of degenerative disorders of the spine, such as cervical spinal stenosis with cervical spine myelopathy (CSM) in the geriatric population, has rapidly increased worldwide. To date, there has been no systematic analysis comparing outcomes in older patients suffering from progressive CSM and undergoing surgery depending on their health insurance status. We sought to compare the clinical outcomes and complications after anterior cervical discectomy and fusion (ACDF) or posterior decompression with fusion in patients aged ≥ 65 years with multilevel cervical spinal canal stenosis and concomitant CSM with special focus on their insurance status.

**Methods:**

Clinical and imaging data were retrieved from patients’ electronic medical records at a single institution between September 2005 and December 2021. Patients were allocated into two groups with respect to their health insurance status: statutory health insurance (SHI) vs. private insurance (PI).

**Results:**

A total of 236 patients were included in the SHI group and 100 patients in the privately insured group (PI) group. The overall mean age was 71.7 ± 5.2 years. Regarding comorbidities, as defined with the age-adjusted CCI, SHI patients presented with higher rates of comorbidities as defined by a CCI of 6.7 ± 2.3 and higher prevalence of previous malignancies (9.3%) when compared to the PI group (CCI 5.4 ± 2.5, *p* = 0.051; 7.0%, *p* = 0.048). Both groups underwent ACDF (SHI: 58.5% vs. PI: 61.4%; *p* = 0.618), and the surgical duration was similar between both groups. Concerning the intraoperative blood transfusion rates, no significant differences were observed. The hospital stay (12.5 ± 1.1 days vs. 8.6 ± 6.3 days; *p* = 0.042) and intenisve care unit stay (1.5 ± 0.2 days vs. 0.4 ± 0.1 days; *p* = 0.049) were significantly longer in the PI group than in the SHI group. Similar in-hospital and 90-day mortality rates were noted across the groups. The presence of comorbidities, as defined with the age-adjusted CCI, poor neurological status at baseline, and SHI status, was significant predictor for the presence of adverse events, while the type of surgical technique, operated levels, duration of surgery, or blood loss was not.

**Conclusions:**

Herein, we found that surgeons make decisions independent of health insurance status and aim to provide the most optimal therapeutic option for each individual; hence, outcomes were similar between the groups. However, longer hospitalization stays were present in privately insured patients, while SHI patients presented on admission with poorer baseline status.

## Introduction

Degenerative forms of cervical spinal myelopathy (CSM) are the most common cause of spinal impairment in the older population, with its prevalence steadily increasing in individuals aged ≥ 50 years. The symptoms are insidious and gradually worsen, leading to diagnosis when patients experience higher grades of disability [2]. Surgery, including surgical decompression, such as posterior or anterior approaches, is considered the gold standard for treating the associated neurological deficits [7, 8, 18, 22]. Therefore, shorter wait times for spine surgery, especially in cases with recently diagnosed neurological deficits, are associated with substantially improved outcomes, especially for older patients [7, 17, 22].

Concordantly, all healthcare systems aim to provide equal access to healthcare to everyone, including older patients with a poor baseline reserve requiring surgery for progressive CSM. However, even in Germany, inequalities are observed depending on the insurance status [12, 15]. For example, patients with a higher socioeconomic status experience shorter wait times in outpatient and inpatient care, are more commonly treated by specialists, and utilize preventive care more frequently [19]. Counterintuitively, privately insured patients (only 10% of all insured individuals) were reported to have a higher chance of being treated by innovative drugs or even be classified with higher urgency for organ transplants compared with their publicly insured counterparts [12, 15, 19].

Collectively, there is a lack in systematic analysis comparing outcomes in older patients with progressive CSM and undergoing surgery depending on their health insurance status. Considering the unique requirement for this cohort owing to the concomitant frailty, we hypothesized that health insurance status might have a significant impact on surgical decision-making and clinical outcomes.

Therefore, we aimed to compare the patient history, clinical and surgical characteristics, clinical outcomes, complications, and morbidity and mortality rates in patients aged ≥ 65 years who underwent anterior cervical discectomy and fusion (ACDF) or posterior decompression with fusion (PDF) for multilevel cervical spinal canal stenosis and concomitant CSM, with particular focus on their health insurance status (social health insurance [SHI] vs. private insurance [PI]).

## Methods

Clinical and imaging data of patients were retrospectively extracted from electronic medical records in our institution’s database between September 2005 and December 2020. The present study was conducted in accordance with the Declaration of Helsinki and approved by the local ethics committee (approval no. S-723/2017). Due to the retrospective nature of this study, the requirement for informed consent was waived. Patients aged ≥ 65 years with cervical canal stenosis and concomitant CSM of at least two levels without signs of instability were consecutively enrolled. Radiological evaluation consisted of radiographs in the standing upright flexion/extension produced by computed tomography (CT) and magnetic resonance imaging (MRI). The inclusion criteria were as follows: symptomatic CSM with at least one clinical sign of myelopathy, at least two levels of the extension of CSM, radiographic imaging-based evidence of cervical spinal cord compression on MRI or CT, and no previous cervical spine surgery. Patients who had myelopathy due to spondylosis, hypertrophy of the ligamentum flavum, OPLL, disc herniation, subluxation, or a combination of these degenerative changes were included in this study. Patients’ insurance status was also extracted from the medical records (SHI vs. PI). The exclusion criteria were as follows: asymptomatic patients; those diagnosed with an active infection, neoplastic disease, spinal instability and progressive kyphotic deformity, rheumatoid arthritis, ankylosing spondylitis; or those without the requisite data.

### Patient population

Patient demographics, comorbidities, American Society of Anesthesiologists (ASA) scores, surgical characteristics, peri- and postoperative complications, hospital length of stay (LOS), intensive care unit (ICU) stay, readmissions, reoperations, and mortality were retrieved from patients’ medical records. Comorbidities present before surgery were assessed using the age-adjusted Charlson Comorbidity Index (CCI) [5, 11]. The CCI was calculated for each patient and classified as follows: no comorbidity (CCI = 0), minimal comorbidity (CCI = 1 or 2), moderate comorbidity (CCI = 3–5), or severe comorbidity (CCI > 5). The severity of the CSM was evaluated before and after surgery according to Modified Japanese Orthopaedic Association (mJOA) score for cervical myelopathy [3]. Postoperative mJOA was documented according to the last clinical and imaging follow-up examination. According to our institutional standards, routine clinical and radiological follow-up examinations were performed before discharge and 3 months after surgery. The final follow-up period was between 3 and 48 months after surgery.

### Procedures

Patients were allocated into one of the following two groups: patients with (1) SHI and (2) PI. All patients underwent ACDF or PDF. Decision-making regarding operative intervention was guided by a multitude of healthcare professionals, including neurosurgeons, neuroradiologists, and anesthesiologists in accordance with the clinical needs of each patient. The final decision was made by an experienced attending spine surgeon. All instrumented surgeries were performed using a CT-based point-to-point navigation system to enhance maximal safety, as described previously by our study group [13].

### Statistical analysis

Categorical variables are presented as numbers and percentages. Continuous variables are presented as means ± standard deviations and were verified as normally distributed using the Shapiro–Wilk test. Baseline and surgical characteristics, readmissions, reoperations, and mortality rates were compared groupwise using independent *t*-tests for continuous variables and chi-square tests for categorical variables. A binary logistic regression analysis was performed to define the potential risk factors for the occurrence of complications. Statistical significance was set at *p* < 0.05. All statistical analyses were performed using SPSS software, version 29.0.0.0 (IBM Corp., Armonk, NY, USA).

## Results

### Demographics and baseline characteristics

Over a period of 16 years, 336 patients aged ≥ 65 years diagnosed with cervical stenosis and concomitant CSM were examined. A total of 236 patients were included in the SHI group and 100 patients in the privately insured group (PI) group. The overall mean age was 71.7 ± 5.2 years. Regarding comorbidities, as defined with the age-adjusted CCI, SHI patients presented with higher rates of comorbidities as defined by a CCI of 6.7 ± 2.3 and higher prevalence of previous malignancies (9.3%) when compared to the PI group (CCI 5.4 ± 2.5, *p* = 0.051; 7.0%, *p* = 0.048). The neurological condition, as defined with the mJOA score, was similar between both groups. A detailed description of baseline characteristics is delineated in Table 1.

**Table 1:** Baseline patient characteristics

	SHI *n* = 236	Private *n* = 100	*p*-value
Age, mean (SD), years	75.8 (5.5)	67.5 (5.1)	**0.002**
Sex, *n* (%)			0.185
Male	136 (57.6)	66 (65.3)	
Female	100 (42.4)	35 (34.7)	
BMI, mean (SD), kg/m^2^	28.0 (6.3)	22.1 (3.2)	**0.044**
Comorbidities			
Age-adjusted CCI score, mean (SD)	6.7 (2.3)	5.4 (2.5)	**0.051**
Arterial hypertension, *n* (%)	91 (38.6)	41 (40.6)	0.726
Myocardial infarction, *n* (%)	39 (16.5)	20 (19.8)	0.468
Coronary heart disease, *n* (%)	43 (18.2)	23 (22.8)	0.335
Atrial fibrillation, *n* (%)	32 (13.6)	20 (19.8)	0.146
Peripheral vascular disease, *n* (%)	28 (11.9)	11 (10.9)	0.798
COPD, *n* (%)	18 (7.6)	11 (10.9)	0.328
Type 2 diabetes mellitus, *n* (%)	42 (17.8)	19 (18.8)	0.824
Renal failure, *n* (%)	28 (11.9)	13 (12.9)	0.796
Liver disease, *n* (%)	13 (5.5)	11 (10.9)	0.078
Gastrointestinal ulcer, *n* (%)	12 (5.1)	8 (7.9)	0.728
TIA/stroke, *n* (%)	8 (3.4)	2 (2.0)	0.485
Malignancy, *n* (%)	22 (9.3)	7 (7.0)	**0.048**
Dementia, *n* (%)	7 (3.0)	2 (2.0)	0.607
Previous spinal surgery	59 (25.0)	17 (16.8)	0.100
Active smoking, *n* (%)	2 (0.8)	10 (10.0)	0.894
ASA class, mean (SD)			0.279
I	3 (1.3)	7 (7.0)	
II	94 (39.8)	49 (48.5)	
III	122 (51.7)	43 (42.6)	
IV	4 (1.7)	2 (2.0)	
mJOA score, mean (SD)	11.1 (1.9)	11.0 (1.7)	0.187

Bold values are considered significant (*p* ≤ 0.050)

Abbreviations: *SHI*, statutory health insurance; *ACDF*, anterior cervical discectomy fusion; *PDF*, posterior decompression and fusion; *ASA*, American Society of Anesthesiology; *BMI*, body mass index; *CCI*, Charlson comorbidity index; *COPD*, chronic obstructive pulmonary disease; *TIA*, transient ischemic stroke; *SD*, standard deviation; *mJOA*, modified Japanese Orthopaedic Association

### Surgical characteristics and clinical course

As displayed in Table 2, both groups underwent ACDF (SHI: 58.5% vs. PI: 61.4%; *p* = 0.618), and the surgical duration was similar between both groups. Concerning the intraoperative blood transfusion rates, no significant differences were observed. The LOS (12.5 ± 1.1 days vs. 8.6 ± 6.3 days; *p* = 0.042) and ICU stay (1.5 ± 0.2 days vs. 0.4 ± 0.1 days; *p* = 0.049) were significantly longer in the PI than in the SHI group. Similar in-hospital and 90-day mortality rates were noted across the groups ranging between 1.3 and 5.0%. Furthermore, the postoperative neurological conditions and readmission rates did not differ between the two groups. Notably, both groups recovered with similar favorable rates, as defined by the mJOA (SHI: 12.3 ± 1.8 vs. PI: 12.9 ± 2.2; *p* = 0.141). Overall, the mean follow-up was 41.2 ± 5.1 months and no additional surgery was necessary due to material failure, screw loosening, or secondary instability during the follow-up period. However, in-hospital revision was performed in two patients (one from the SHI and one from the PI group) in the posterior decompression and instrumentation group due to screw loosening after PDF, while two patients (one from the SHI and one from the PI group) required revision because of epidural hematoma.

**Table 2:** Comparison of surgical characteristics and clinical course between groups

Characteristic	SHI *n* = 236	Private *n* = 100	*p*-value
Surgical duration, mean (SD), min	165.3 (83.1)	170.3 (82.3)	0.568
Surgical approach, *n* (%)			0.618
ACDF	138 (58.5)	62 (61.4)	
PDF	98 (41.5)	39 (38.6)	
No. of levels decompressed/fused	2.3 (1.1)	2.4 (0.9)	0.721
Surgical blood loss, mean (SD), ml	134.9 (133.1)	140.5 (132.4)	0.556
Intraoperative blood transfusion, *n* (%)	4 (1.7)	3 (3.0)	0.457
Length of stay, *n* (%), days	8.6 (6.3)	12.5 (1.1)	**0.042**
ICU stay, mean (SD), days	0.4 (0.1)	1.5 (0.2)	**0.008**
Mortality			
In-hospital mortality, *n* (%)	3 (1.3)	3 (3.0)	0.280
90-day mortality, *n* (%)	4 (1.7)	5 (5.0)	0.060
30-day readmission, *n* (%)	3 (1.3)	2 (2.0)	0.382
Post-mJOA score, mean (SD)	12.3 (1.8)	12.9 (2.2)	0.141

Bold values are considered significant (*p *≤ 0.050)

Abbreviations: *SHI*, statutory health insurance; *ACDF*, anterior cervical discectomy fusion; *PDF*, posterior decompression and fusion; *ICU*, intensive care unit; *MS*, motor score; *SD*, standard deviation; *mJOA*, modified Japanese Orthopaedic Association

### Complications and associated risk factors

No significant differences were observed in the occurrence of postoperative complications between the groups. A detailed breakdown of all the recorded complications is provided in Table 3. In the second stage analysis, logical regression models were applied to determine the potential risk factors for the occurrence of complications. The presence of comorbidities, as defined with the age-adjusted CCI, poor neurological status at baseline, and SHI status, was significant predictor for the presence of adverse events, while the type of surgical technique, operated levels, duration of surgery, or blood loss were not (Table 4).

**Table 3:** Occurrence of adverse events and revision rates

	SHI *n* = 236	Private *n* = 100	*p*-value
Superficial wound infection, *n* (%)	6 (2.5)	4 (4.0)	0.482
Acute heart failure, *n* (%)	6 (2.5)	1 (1.0)	0.360
Thrombotic event, *n* (%)	0 (0.0)	1 (1.0)	0.126
Pneumonia, *n* (%)	8 (3.4)	2 (2.0)	0.485
Dysphagia, *n* (%)	4 (1.7)	3 (3.0)	0.452
Ileus, *n* (%)	5 (2.1)	1 (1.0)	0.473
Revision surgery, *n* (%)			
Epidural hematoma	1 (1.7)	1 (1.0)	0.452
Screw loosening	0 (0.0)	1 (1.0)	0.492

Abbreviations: *SHI*, statutory health insurance; *ACDF*, anterior cervical discectomy fusion; *PDF*, posterior decompression and fusion

**Table 4:** Risk factors for complications

Complications	OR (95% CI)	*p*-value
Age-adjusted CCI score	2.5 (1.1–3.9)	**0.025**
SHI (Insurance status ^a^)	1.6 (1.1–2.9)	**0.005**
Preoperative mJOA	1.7 (1.1–3.5	0.040
Fusion surgery^b^	0.5 (0.2–0.9)	0.650
Operated segments	1.9 (0.5–5.5)	0.322
Duration of surgery	1.2 (0.9–1.7)	0.789
Blood loss	1.1 (0.8–1.2)	0.573

Abbreviations: *OR*, odds ratio; *CI*, confidence interval; *CCI*, Charlson comorbidity index

^a^Reference, PI (private insurance); SHI statutory health insurance

^b^Reference, PDF, posterior decompression and fusion

The *p*-values presented in bold font indicate statistically significant results

## Discussion

To the best of our knowledge, this is the first study investigating surgical strategies for the therapy of degenerative cervical canal stenosis with CSM in patients aged 65 years and older with respect to their insurance status. In this study, we demonstrated that patients with SHI were significantly older with higher grades of comorbidities, as defined by a CCI score of 6.7 and the prevalence of obesity compared with their privately insured counterparts. Notably, patients with SHI had a higher prevalence of cancer diseases. We did not observe any significant differences in the choice of surgical approach or surgical characteristics. Both cohorts exhibited neurological improvement after surgery. Notably, privately insured patients had significantly longer intensive care unit (ICU) and hospital stays, whereas the mortality rates were similar between the groups. Notably, SHI status, higher grades of comorbidities, and worse neurological status at baseline were unique risk factors for the occurrence of complications, whereas the surgical technique or duration was not. To the best of our knowledge, this is the first study investigating surgical strategies for the therapy of degenerative cervical canal stenosis with CSM in patients aged ≥ 65 years with respect to the insurance status.

Older patients present with a poor baseline reserve; thus, this subset of patients is at an increased risk after surgical procedures, especially those performed for the management of spinal pathologies [8, 10]. In the German healthcare system, individuals with private insurance generally undergo health checks before obtaining insurance and are often healthier compared to those with statutory insurance. Statutory insurance, which provides coverage for all citizens irrespective of their health status, is likely to include a higher proportion of individuals with severe or multiple comorbidities [24]. Our study aimed to understand the potential influence of insurance status on postoperative complications, taking into account the pre-existing comorbidities among the patient population. Importantly, our analyses controlled for the presence and severity of comorbidities, aiming to separate their effect from that of the insurance status. Interestingly, our data indicated that privately insured patients experienced significantly fewer postoperative complications. This suggests that the lower risk of postoperative complications in privately insured patients could be partially attributable to their lower burden of comorbidities, indicating a potentially influential role of insurance status in postoperative outcomes. Nevertheless, we concur with your assertion that the severity of comorbidities is a key predictor of postoperative complications, and it should always be considered paramount in clinical decision-making. The association between insurance status and health outcomes should be interpreted judiciously, acknowledging that multiple factors, such as access to healthcare, quality of care, and overall health status, interact intricately to influence these outcomes[16, 20]. Further research is needed to gain deeper insight into these relationships and to inform strategies for improving patient outcomes irrespective of insurance status.

CSM is the most common type of spinal cord dysfunction in patients aged ≥ 55 years and gradually leads to neurological deficits, such as gait disturbance or paraparesis. Despite several reports on this topic [2, 7, 9, 10], we lack consensus on the most efficient treatment approach (anterior vs. posterior) for such patients, especially those with advanced age. A well-accepted premise is that anterior approaches, such as ACDF, are mainly utilized for anteriorly located osteophytes, ossification of the posterior ligament, disc herniation, or when one or two levels are involved. In contrast, posterior decompression and fixation offer a wider decompression, especially in cases with maximal cervical stenosis overwhelmingly seen in the older population [1]. However, the influence of the health insurance status of patients on the decision-making and surgical procedures preferred based on costs remains unexplored. Notably, our findings confirmed that the surgical procedures were guided by the pathology and needs of each individual. Herein, it should be considered that PDF is associated with increased costs compared to ACDF [4]. Kings et al., in their retrospective study on PDF vs. ACDF based on claim data, reported that PDF results in increased hospital charges ($23,400 vs. $14,300) and longer length of stay (LOS; 4.6 vs. 3.8 days) compared with ACDF [14]. Concordantly, Tanenbaum et al. assessed data from a National Inpatient Sample and reported that PDF is associated with approximately 99.4% higher in-hospital charges and 6.5 days longer LOS. In contrast, ACDF costs were approximately $40,000 lesser, and the LOS was 2 days shorter [21]. However, a major limitation of the aforementioned studies is that their cost analysis did not consider insurance status; hence, the impact of insurance status on decision-making could not be detected. Moreover, the patient sample consisted of individuals of all age groups; therefore, potential relationships between the baseline characteristics and the surgical procedure remained marginal. In our present study, we demonstrated that the decision-making of surgeons is guided by pathology (clinically and radiologically) and not the insurance status of the patients; however, we speculate that private insurance contributed to longer hospitalization stays.

In the German healthcare system, PI patients are billed based on a fee-for-service model, which operates in accordance with the German Medical Fee Schedule (Gebührenordnung für Ärzte, GOÄ) or the Hospital Fee Schedule (Gebührenordnung für Krankenhausleistungen, GOP). Under this model, charges are applied for every individual service a patient receives, ranging from medical consultations to diagnostic procedures, therapeutic interventions, and ancillary services such as nursing care and nutritional counseling. Research, including a study by Lungen et al. (2008), suggests that privately insured patients may receive a broader scope of these services compared to their publicly insured counterparts [24]. Consequently, this comprehensive service provision may result in longer hospital stays for privately insured patients. Hospitals may indeed have a financial incentive to provide more services and extend stays for these patients, given the higher reimbursement rates from private insurers under the fee-for-service model. However, it is crucial to stress that clinical and surgical decision-making should always prioritize patient health outcomes over financial considerations. The delivery of healthcare services, including the determination of the length of hospital stay, should be guided by the medical needs of the patient rather than their insurance status [6] Despite the potential for increased revenue from privately insured patients, healthcare providers have an ethical obligation to deliver the same high standard of care to all patients, regardless of their insurance status [23].

In conclusion, while privately insured patients may stay longer in hospital and potentially receive more services, leading to higher charges, this should not influence the quality of care provided or decisions regarding their care. Patient well-being should always remain the driving factor in all healthcare decisions.

While the ethical impetus for surgeons is to base decisions strictly on the medical condition and the best interest of the patient, it is recognized that extraneous factors can inadvertently influence their judgement. Understanding the potential ramifications of insurance status on clinical decision-making is vital to dissect healthcare disparities and confront emergent biases.

The fundamental premise of this study was to interrogate the potential influence of insurance status on surgical decision-making. Nevertheless, it is crucial to underscore the context of our methods: decision-making regarding surgical interventions involved a comprehensive interdisciplinary discussion. This encompassed insights from healthcare professionals such as neurosurgeons, neuroradiologists, and anesthesiologists, who considered the clinical requirements of each patient. Ultimately, an experienced attending spine surgeon made the final determination. Notably, many of the patients in this study presented with comorbidities. Consequently, a detailed interdisciplinary evaluation was imperative to gauge the feasibility and associated risks of surgical intervention. The final decision regarding the type of surgery hinged on a multitude of factors, primarily encompassing the patient’s clinical status and a risk–benefit analysis of the procedure. In the context of the legal and ethical guidelines that govern medical practice in Germany, the Federal Ministry of Health has enshrined principles that ensure the patient’s insurance status does not factor into clinical decision-making or the course of treatment. The Patient Rights Law, in particular, advocates for patient autonomy, informed decision-making, and equitable access to healthcare services. These principles not only underscore the ethos of our study but also are intrinsically woven into the decision-making process within our methodology. Our study, while exploring the potential ramifications of insurance status on surgical decisions, maintains its fidelity to the overarching principle of delivering healthcare based on clinical needs. This assertion remains steadfastly true regardless of the insurance status of the patient. Our findings reflect the commitment of German legislation and the healthcare system to provide high-quality, equitable medical care for all citizens. In essence, the hypothesis posed and the results garnered from this study should be viewed through the lens of this foundational principle of German healthcare. Regardless of the insurance status, the most suitable therapeutic options are provided to each patient in alignment with German law, thus substantiating our commitment to fair and individualized healthcare.

Surgeons and other healthcare professionals regularly grapple with complex ethical dilemmas in their practices, aiming to deliver care based on medical necessity and patient well-being. This task becomes more intricate when potential external factors like insurance status may influence decision-making, particularly in settings where healthcare resources may be limited.

Over the past decade and a half, transformative changes in both the statutory and private healthcare insurance sectors in Germany may have had ramifications on patient outcomes. The statutory insurance landscape witnessed the introduction of a centralized health fund in 2009, a reform purposed to bolster competition and financial stability [1, 7] Concurrently, the private insurance sector underwent revisions aiming to improve the accessibility and affordability of healthcare, specifically for individuals with pre-existing conditions or those of advanced age [1, 7]. Considering these amendments, their potential impact on the findings of our study must be acknowledged. Changes in the statutory health insurance, with the introduction of the central health fund, potentially led to enhanced equity in healthcare access. This could have resulted in improved health outcomes for patients covered by statutory health insurance, as better access to healthcare services can contribute to early disease detection and treatment. Conversely, reforms in the private insurance sector primarily targeted to make private healthcare more accessible and affordable may have broadened the spectrum of insured individuals, potentially including those with higher health risks. This development could lead to a shift in the risk profile of privately insured patients over time, which might affect the observed association between private insurance status and postoperative complications in our study. It is worth noting that these speculations are based on the macroscopic changes in health insurance policies over the years and might not directly mirror individual patient experiences or account for all confounding factors. A more granular analysis, perhaps considering individual patient data over time, would be needed to draw more definite conclusions.

### Limitations

The main strength of the current study is that it is the first study to examine surgical outcomes in a large cohort of older patients undergoing spinal ACDF versus PDF with respect to health insurance status. However, this study had some limitations. First, this was a retrospective study, and selection bias may have been present. Nevertheless, considering the vulnerability of this subset of patients, we believe that the present number of patients may generate a real-world picture of the optimal treatment of this condition with respect to insurance status. The number of severely ill patients might be small; however, owing to the vulnerability of this population, we believe that the acquired number of patients exhibited significant statements of the treatment algorithms with a special focus on insurance status. A cost-effectiveness analysis might have been useful; however, our findings demonstrate that decision-making was determined by the unique requirement of the patient and not their health insurance status.

## Conclusions

As life expectancy increases due to healthcare advancements, the demand for surgical interventions is expected to rise. Our study highlights that surgeons base their decisions on medical necessity, irrespective of insurance status, resulting in similar outcomes for both privately and statutorily insured patients. Consequently, further research is needed to better understand these subjective influences and address potential healthcare disparities.

## Data Availability

The datasets generated during and/or analyzed during the current study are available from the corresponding author on reasonable request.
